# The effects of bariatric surgery on clinical profile, DNA methylation, and ageing in severely obese patients

**DOI:** 10.1186/s13148-019-0790-2

**Published:** 2020-01-20

**Authors:** Eliza Fraszczyk, Mirjam Luijten, Annemieke M. W. Spijkerman, Harold Snieder, Paul F. K. Wackers, Vincent W. Bloks, Carolina F. Nicoletti, Carla B. Nonino, Ana B. Crujeiras, Wim A. Buurman, Jan Willem Greve, Sander S. Rensen, Bruce H. R. Wolffenbuttel, Jana V. van Vliet-Ostaptchouk

**Affiliations:** 1 Department of Epidemiology, University of Groningen, University Medical Center Groningen, Groningen, The Netherlands; 2 Centre for Health Protection, National Institute for Public Health and the Environment (RIVM), Bilthoven, The Netherlands; 3 Centre for Nutrition, Prevention and Health services, National Institute for Public Health and the Environment (RIVM), Bilthoven, The Netherlands; 4 Department of Pediatrics, section of Molecular Metabolism and Nutrition, University of Groningen, University Medical Center Groningen, Groningen, The Netherlands; 5 Laboratory of Nutrigenomics Studies, Department of Internal Medicine, Ribeirão Preto Medical School, University of Sao Paulo, Sao Paulo, Brazil; 6 Laboratory of Nutrigenomics Studies, Department of Health Sciences, Ribeirão Preto Medical School, University of Sao Paulo, Sao Paulo, Brazil; 7 Epigenomics in Endocrinology and Nutrition, Health Research Institute of Santiago (IDIS), University Clinical Hospital of Santiago (CHUS/SERGAS) and Santiago de Compostela University (USC), Santiago de Compostela, Spain; 8 CIBER Fisiopatologia de la Obesidad y Nutricion (CIBERobn), Madrid, Spain; 9 School for Mental Health and Neuroscience, Maastricht University, Maastricht, The Netherlands; 10 Department of Surgery, Zuyderland Medical Center Heerlen, Dutch Obesity Clinic South, Heerlen, The Netherlands; 11 Department of Surgery, Maastricht University Medical Center, NUTRIM School for Nutrition and Translational Research in Metabolism, Maastricht, The Netherlands; 12 Department of Endocrinology, University of Groningen, University Medical Center Groningen, Groningen, The Netherlands; 13 Genomics Coordination Center, Department of Genetics, University of Groningen, University Medical Center Groningen, Hanzeplein 1, 9713 GZ Groningen, The Netherlands

**Keywords:** Obesity, Bariatric surgery, Morbid obesity, DNA methylation, Epigenetics, Epigenetic clock, Biological age, EWAS

## Abstract

**Background:**

Severe obesity is a growing, worldwide burden and conventional therapies including radical change of diet and/or increased physical activity have limited results. Bariatric surgery has been proposed as an alternative therapy showing promising results. It leads to substantial weight loss and improvement of comorbidities such as type 2 diabetes. Increased adiposity is associated with changes in epigenetic profile, including DNA methylation. We investigated the effect of bariatric surgery on clinical profile, DNA methylation, and biological age estimated using Horvath’s epigenetic clock.

**Results:**

To determine the impact of bariatric surgery and subsequent weight loss on clinical traits, a cohort of 40 severely obese individuals (BMI = 30–73 kg/m^2^) was examined at the time of surgery and at three follow-up visits, i.e., 3, 6, and 12 months after surgery. The majority of the individuals were women (65%) and the mean age at surgery was 45.1 ± 8.1 years. We observed a significant decrease over time in BMI, fasting glucose, HbA1c, HOMA-IR, insulin, total cholesterol, triglycerides, LDL and free fatty acids levels, and a significant small increase in HDL levels (all *p* values < 0.05). Epigenome-wide association analysis revealed 4857 differentially methylated CpG sites 12 months after surgery (at Bonferroni-corrected *p* value < 1.09 × 10^−7^). Including BMI change in the model decreased the number of significantly differentially methylated CpG sites by 51%. Gene set enrichment analysis identified overrepresentation of multiple processes including regulation of transcription, RNA metabolic, and biosynthetic processes in the cell. Bariatric surgery in severely obese patients resulted in a decrease in both biological age and epigenetic age acceleration (EAA) (mean = − 0.92, *p* value = 0.039).

**Conclusions:**

Our study shows that bariatric surgery leads to substantial BMI decrease and improvement of clinical outcomes observed 12 months after surgery. These changes explained part of the association between bariatric surgery and DNA methylation. We also observed a small, but significant improvement of biological age. These epigenetic changes may be modifiable by environmental lifestyle factors and could be used as potential biomarkers for obesity and in the future for obesity related comorbidities.

## Background

Worldwide prevalence of obesity has nearly tripled since 1975. In 2016, 39% of the adult population were overweight (defined as body mass index (BMI) ≥ 25) and 13% were obese (defined as BMI ≥ 30) making obesity a global burden [1]. Obesity is recognized as one of the major risk factors for chronic diseases like cardiovascular disease and type 2 diabetes, as well as cancer and depression [2]. It is often accompanied by chronic, low grade inflammation, high levels of pro-inflammatory cytokines, and low levels of anti-inflammatory adipokines [3].

Conventional treatment of severe obesity includes intensive lifestyle modifications such as a radical change in diet and/or increased physical activity [4]. However, those interventions are often insufficient, while the possibilities for pharmacological treatment are limited [5, 6]. Recently, bariatric surgery has been introduced as an alternative therapy. This involves a variety of surgical procedures, which results in weight loss and subsequent improvement of obesity-related co-morbidities [7]. Long-term studies have shown that bariatric surgery can reduce hyperglycemia or even introduce diabetes remission in obese patients with type 2 diabetes [8]. Such metabolic improvement has been observed even before weight loss has occurred [9]. The mechanisms underlying metabolic improvement after bariatric surgery are not fully understood yet, but epigenetic factors have been proposed to play a significant role [10, 11]. This is because the obesity-related metabolic disturbances are produced as a result of an interaction between environmental, lifestyle, and genetic factors and epigenetics mediates the environmental effect on the cellular function of the organism [12]. One of the most studied epigenetic mechanisms is DNA methylation, which includes methyl group binding to the cytosines of cytosine-guanine dinucleotides (CpG sites) [13]. It has been shown that DNA methylation can be influenced by environmental and lifestyle factors, including smoking and unhealthy diet [14, 15]. Also, alterations in DNA methylation levels have been shown to be a consequence of increased adiposity [16] and this epigenetic mechanism was proposed as a potential link between obesity and its comorbidities such as insulin resistance [17] and cancer [18, 19]. Therefore, we hypothesized that weight loss resulting from bariatric surgery may lead to changes in DNA methylation profiles, potentially providing insight into molecular mechanisms underlying weight loss–induced metabolic improvement.

Together with obesity, aging is a risk factor for many chronic diseases [20]. A recently developed biological age estimator based on DNA methylation known as epigenetic clock has been repeatedly shown to be associated with many age-related conditions such as cancer, neurodegeneration, and menopause [21–23], but also with BMI and obesity [20, 24]. The difference between DNA methylation age, also referred to as biological age, and chronological age is called age acceleration. Positive or negative values of epigenetic age acceleration (EAA) suggest that a specific tissue is aging faster or slower than expected. To date, EAA has been associated with level of education, diet, and other lifestyle factors [25]. Here we hypothesized that EA, which represents biological age, is higher in severely obese patients before than after bariatric surgery.

The present study aims to investigate the effect of bariatric surgery on (a) anthropometric, metabolic, and lipid changes at three time points after surgery (3, 6, and 12 months); (b) changes in DNA methylation by comparing methylation profiles in peripheral blood just before and 12 months after surgery; (c) biological age and epigenetic age acceleration (EAA) by comparing these just before surgery and 12 months after the surgery.

## Methods

### Study population

A group of 40 severely obese patients underwent elective bariatric surgery (See Additional file 1: Table S1 for details of the surgery) at the Department of General Surgery, Maastricht University Medical Center (Maastricht, the Netherlands). We included baseline and follow-up data (3, 6, and 12 months after the surgery) for these individuals. Details of the study can be found elsewhere [26, 27]. This study was approved by the Medical Ethics Board of Maastricht University Medical Centre, in line with the ethical guidelines of the 1975 Declaration of Helsinki. Informed consent was obtained from each participant.

### Measurements

A standardized protocol was used to obtain blood pressure and anthropometric measurements such as height, weight, and waist circumference. Blood samples were collected after 8 h fasting on the morning of surgery for determining fasting blood glucose, HbA1c, insulin, homeostatic model assessment (HOMA-IR), total cholesterol, HDL cholesterol, LDL cholesterol, triglycerides (TG), free fatty acids (FFA), alanine transaminase (ALAT), aspartate transaminase (ASAT), and C-reactive protein (CRP).

DNA was isolated from whole blood samples collected at two time points, i.e., on the morning of surgery and 12 months after surgery. A total of 500 ng of genomic DNA was bisulfite converted using the EZ DNA Methylation kit (Zymo Research, Irvine, CA, USA) and hybridized to Illumina 450 K arrays (San Diego, CA, USA) according to the manufacturer’s protocols. Data were generated by the Genome Analysis Facility of UMCG (www.rug.nl/research/genetics/genomeanalysisfacility/), using the same batch of arrays for all study participants.

### DNA methylation quality control and normalization

Handling, analysis, and visualization of the data were performed in R statistical software version 3.5.1 (http://cran.r-project.org). Raw signal intensities were imported in R and subsequently subjected to quality control using the minfi package [28]. These quality checks revealed no major batch effects. The functional normalization method was used to normalize the data using the standard Illumina probe design information (IlluminaHumanMethylation450k array) [29]. Additional probe filtering was performed to remove from the dataset probes containing a SNP in the probes sequence, probes with a poor detection *p* value (*p* > 0.001), and probes on X and Y chromosomes. We checked for the presence of extreme outliers in the methylation data (< 25th percentile − 3IQR (interquartile range) or > 75th percentile + 3IQR) as suggested by the PACE consortium [30]. This resulted in the removal of 19 additional probes (if outliers were detected in > 20% of samples), and setting all identified outliers to missing values. The final dataset for analysis included 456,073 probes.

### Statistical analysis

#### Clinical changes

Linear mixed models were used to calculate the overall significance of clinical changes over time since baseline, with a random intercept, random coefficient for time, and an unstructured covariance matrix. CRP and TG levels were log-transformed since these parameters did not show normal distribution. All analysis were adjusted for sex and age differences and calculated using the “lme4” package in R.

#### Epigenome-wide association analysis

To find differences in DNA methylation levels within the same subject over two time points (before and 12 months after bariatric surgery) we used the limma package for microarray experiments [31]. Linear regression model with individual as a random effect was applied. We adjusted our analysis for cell type distribution, estimated at 2 time points, before and after surgery, using Houseman’s estimation method [32]. We also tested if there was a significant shift in estimated white blood cell distributions pre- and post-surgery using a paired *t* test. Normalized beta values were logit-transformed into M-values for downstream analysis because they have been shown to perform better in smaller studies [33]. As a sensitivity analysis in those with complete covariate data (*n* = 30) we adjusted our main analysis model for BMI, insulin, glucose, and CRP changes pre- and post-surgery. We adjusted all epigenome-wide analyses (EWAS) for multiple testing using Bonferroni correction. We also calculated the Pearson correlation between significant CpG sites from the main analysis and 14 clinical variables, which were measured pre- and post-surgery (waist-hip ratio, BMI, glucose, insulin, HOMA-IR, HbA1c, total cholesterol, HDL cholesterol, LDL cholesterol, TG, FFA, ALAT, ASAT, CRP). We considered a correlation as “moderate to strong” in case *R* > 0.5 and *p* < 0.01.

#### Replication and comparison to the general population

We attempted to replicate our findings in two independent cohorts with similar data [34, 35]. Cohort 1 included 11 severely obese patients (7 women and 4 men) who underwent Roux-en-Y gastric bypass (RYGB) surgery. Details of the study can be found elsewhere [34]. The raw DNA methylation data before and 6 months after the surgery was retrieved from ArrayExpress (https://www.ebi.ac.uk/arrayexpress/) and the same pipeline for quality control, normalization, and EWAS analysis as described above was applied. Cohort 2 included 24 severely obese women, who also underwent RYGB. Normalized DNA methylation beta values and phenotype data before and 6 months after the surgery was obtained from the authors of the study [35]. First, we performed EWAS using the same method as described above. Then, we looked up our significant CpG sites in the replication sample results. Finally, we performed a meta-analysis of all 3 cohorts to find the combined effect of bariatric surgery on DNA methylation. Inverse-variance fixed-effects meta-analyses of single CpG EWAS results were performed using METAL [36].

Additionally, we compared methylation levels at all significant CpG sites with healthy lean and healthy obese people from a general population sample. DNA methylation data from subsets of the Lifelines cohort including 50 healthy lean (BMI < 25) and 50 healthy obese individuals (BMI > 30, without metabolic complications according to the NCEP-ATPIII definition) was available [37]. General details on the Lifelines cohort are described elsewhere [38, 39]. Methylation profiles in those Lifelines groups were measured in the same experiment as those from bariatric surgery patients. We evaluated similarity to the healthy Lifelines groups by comparing the mean methylation levels of all significant CpG sites at pre- and post-surgery with those from the healthy Lifelines groups. We performed a binomial test to determine the significance of similarity between cohorts.

#### Gene set enrichment analysis and association with gene expression

To find functional interpretation of the results, using the significant findings from EWAS as input, we performed gene set enrichment analysis with the “methylGSA” R package [40] to identify significantly enriched Reactome pathways with *p* values for enrichment being adjusted for multiple testing using false discovery rate (FDR < 5%). Additionally, we used the CpG sites that were significantly correlated with clinical traits as input into the Database for Annotation, Visualization and Integrated Discovery (DAVID 6.7; http://david.abcc.ncifcrf.gov/summary.jsp) [41] and the MetaCore analysis tool (GenoGo, Inc.) to find functions of the genes and enrichment of associated diseases. In DAVID, we used gene ontology (GOTERM: BP_FAT), followed by MetaCore overall enrichment analysis including GO processes and enrichment of diseases by biomarkers. Transcription network analysis in MetaCore was applied to identify whether subsets of the genes were regulated by known transcription factors (TFs). Next, we investigated the association between our top significant CpG sites and gene expression levels in blood using publicly available eQTMs from the BIOS consortium (https://www.genenetwork.nl/biosqtlbrowser/).

#### Epigenetic age analysis

Raw beta-values of the 353 age-related CpG sites were used to estimate biological age using the DNA methylation age calculator developed by Horvath [42]. Age acceleration values for both pre- and post-surgery were calculated by subtracting the chronological age from the estimated DNA methylation age, using one tailed paired *t* tests to determine the significance of the difference in age acceleration before and after surgery within the same individual. *p* values below 0.05 were considered significant for these analyses.

## Results

### Clinical traits before and after bariatric surgery

**Fig. 1:**
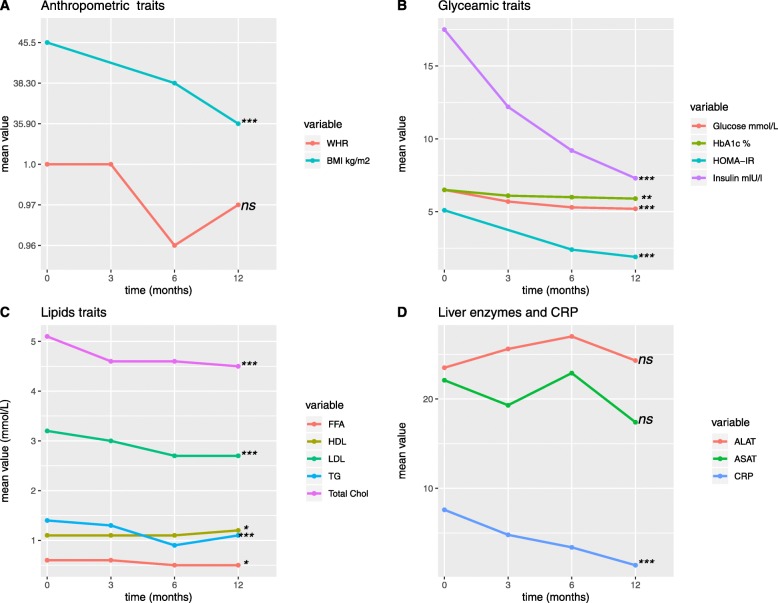
Mean trends over time (at baseline and the three follow-up visits, i.e., 3, 6, and 12 months) in clinical measurements for severely obese patients. **a** Anthropometric traits; **b** glycemic traits; **c** lipids traits; **d** liver enzymes and CRP; presented as mean values of clinical measurements over four time points. Trends over time were calculated using mixed models; *p* values: *ns*, not significant, *< 0.05, **< 0.01, ***< 0.001

To determine the role of bariatric surgery and subsequent weight loss on clinical traits, a cohort of 40 severely obese individuals (BMI > 35 kg/m^2^) was examined at the time of surgery and at three follow-up visits, i.e., 3, 6, and 12 months after surgery. All clinical and anthropometric data collected at baseline and at 3, 6, and 12 months after surgery are presented in Table 1. The majority of individuals were women (65%) and the mean age before the surgery was 45.1 ± 8.06. On average, the levels of many glucose and lipid-related variables, i.e., glucose, HbA1c, HOMA-IR, total cholesterol, TG, and before surgery were elevated compared to blood laboratory reference ranges [43]. Overall mean values of clinical measurements for the total study group at baseline and three follow-up visits are presented in Fig. 1. We observed significantly lower levels over time in BMI, fasting glucose, Hba1c, HOMA-IR, insulin, total cholesterol, TG, LDL, and FFA and a significantly, slightly higher HDL levels. A considerably lower BMI was found 12 months after the bariatric surgery with a mean difference of − 9.57 kg/m^2^ (Table 1). At baseline, significant differences between men and women were found in waist to hip ratio, HDL cholesterol, FFA, and CRP levels (Additional file 1: Table S2, *p* value < 0.05).

**Table 1:** Baseline characteristics of the severely obese cohort who underwent bariatric surgery

	Pre-surgery	3 months after the surgery	6 months after the surgery	12 months after the surgery	Mean difference 12 months-baseline (sd)	*p* value
Sex (females) *n*, %	26 (65%)	26 (65%)	26 (65%)	26 (65%)		
Age (years)	45.1 (8.06)	-	-	46.1 (8.06)		
WHR	1 (0.15)^b^	1 (0.15)	0.96 (0.13)^b^	0.97 (0.13)^a^	− 0.06 (0.13)	0.095
BMI kg/m2	45.5 (9.3)		38.3 (9.2)^a^	35.9 (8.35)	− 9.57 (5.9)	5.1 × 10^−13^
Fasting glucose mmol/L	6.5 (2.2)	5.7 (1.5)	5.3 (1.1)^a^	5.2 (1.2)^b^	− 1.17 (1.8)	1.6 × 10^−4^
Insulin ulU/L	17.5 (10.5)^a^	12.2 (8.7)	9.2 (5.5)^b^	7.3 (4.4)^b^	− 10.7 (8.3)	1.3 × 10^−8^
HOMA-IR	5.1 (3.7)^a^	-	2.4 (1.5)^c^	1.9 (1.1)^c^	− 3.9 (3.7)	9.4 × 10^−7^
HbA1c %	6.5 (1.1)^a^	6.1 (0.9)	6 (0.7)^b^	5.9 (0.9)^b^	− 0.56 (0.8)	0.001
Total cholesterol mmol/L	5.1 (1.1)^a^	4.6 (0.7)	4.6 (0.8)^b^	4.5 (1.2)^b^	− 0.63 (0.8)	2.3 × 10^−4^
HDL mmol/L	1.1 (0.4)^a^	1.1 (0.4)	1.1 (0.4)^b^	1.2 (0.4)^b^	0.16 (0.3)	0.026
LDL mmol/L	3.2 (1)^a^	3.0 (0.8)	2.7 (0.8)^b^	2.7 (1)^b^	− 0.57 (0.8)	4.1 × 10^−4^
TG mmol/L	1.4 [1.0, 2.4]^a^	1.3 [0.9, 1.8]	0.9 [0.8, 1.8]^b^	1.1 [0.7, 1.5]^b^	− 0.5 (0.7)	1.4 × 10^−6^
FFA mmol/L	0.6 (0.3)^b^	0.6 (0.3)	0.5 (0.2)^b^	0.5 (0.2)^b^	− 0.10 (0.34)	0.034
ALAT U/L	23.5 (10.2)^a^	25.6 (10.8)	27 (20.9)^b^	24.3 (10.8)^b^	2.5 (12.1)	0.650
ASAT U/L	22.1 (9.2)^a^	19.3 (6.7)	22.9 (15)^b^	17.4 (7.1)^b^	− 3.7 (11.2)	0.064
CRP mg/L	7.6 [3.2,16.5]^a^	4.8 [1.6, 11.5]	3.4 [1.7,5.5]^c^	1.4 [1, 2.9]^b^	− 4.8 (10)	8.8 × 10^−7^

Data shown as mean (sd) for normally distributed variables, as median [25%, 75%] for not normally distributed variables and as *n* (%) for categorical variables, *p* value is calculated using mixed models

^a^1–5 NA’s

^b^6–10 NA’s

^c^ > 10 NA’s

In this study population, the mean levels of CRP decreased steadily after the intervention, suggesting a decrease in chronic inflammation status (Fig. 1, Table 1). 37.5% of the individuals included in the study had diabetes at the pre-surgical state. We observed an improvement in hyperglycemia reflected by significant reduction in fasting glucose (mean decrease − 1.17 mmol/L) and HbA1c (mean decrease − 0.6 %) 12 months after surgery (*p* value trend over time < 0.001; Table 1). The HOMA-IR index of insulin resistance and β-cell function was improved after the surgery (mean decrease − 3.9; *p* value trend over time = 9.4−10^−7^, Table 1).

### DNA methylation before and after bariatric surgery

#### EWAS analysis

DNA methylation analysis of peripheral blood was conducted for two time points, i.e., before and 12 months after surgery. Epigenome-wide association analysis revealed 4857 significantly differentially methylated CpG sites 12 months after surgery (at Bonferroni-corrected *p* value < 1.09 × 10^−7^; Additional file 1: Table S3; model adjusted for age, sex, cell type distribution, and batch effects). All significant CpG sites showed higher methylation levels after surgery (mean beta change from 0.01% up till 15%; Additional file 2: Figure S1) and were distributed across all chromosomes (Additional file 2: Figure S2). Among the significantly differentially methylated CpG sites, we identified significant enrichment of CpG sites located in gene promoter regions, in 5’UTR and in the 1st exon, but also in CpG islands compared to the total array (all *p* values < 2.2 × 10^−16^ based on Fisher’s exact test; Additional file 2: Figure S3).

Estimation of the white blood cell type distribution using the method by Houseman [32] revealed a clear shift from pre-surgery to post-surgery. This shift, shown in Additional file 2: Figure S4, was statistically significant (*p* value < 0.01) for all estimated cell types, including granulocytes, monocytes, B cells, NKs, CD8T’s, and CDT4’s.

#### EWAS sensitivity analysis

As a sensitivity analysis, we adjusted our main model for changes in BMI, insulin, glucose, and CRP (12 months after the surgery time point versus the baseline), using a subset of 30 samples with complete covariate data. We found 3649 significant CpG sites in the base model adjusted for cell types. After separate adjustments for change in single traits, we observed a decrease of 6–54% in the number of significant CpG sites associated with bariatric surgery, with the largest effects of BMI and insulin. Adding all covariates in a single model decreased the number of significant CpG sites by 62% (Table 2, Venn diagram in Additional file 2: Figure S5).

**Table 2:** Number of significant differentially methylated CpG sites before and after the surgery before and after adjustment for clinical variables (*n* = 30).

Model	Number of Bonferroni corrected significant CpG sites (% decrease from model 1)
Model 1 (adjusted for cell type distribution)	3649
Model 1 + BMI	1813 (51%)
Model 1 + insulin	1686 (54%)
Model 1 + CRP	3294 (10%)
Model 1 + glucose	3415 (6%)
Model 1 + BMI+ insulin + CRP + glucose	1386 (62%)

#### Correlations between post-surgery epigenetic changes and clinical profile

In order to decipher presumably clinically relevant CpG sites, we calculated correlations between DNA methylation change on 4857 significant CpG sites and changes in clinical traits. We found 420 unique significant correlations between CpGs and post-bariatric changes (correlation coefficient *R* > 0.5, *p* < 0.01), among which 33 CpG sites correlated with more than one trait (Additional file 1: Table S4).

#### Gene Set Enrichment analysis

Gene set enrichment analysis, as one of the possible types of post-EWAS analysis, has previously been shown to help functionally interpret the results of genome-wide association studies (GWAS) [44]. Among the 4857 significant CpG sites, we identified overrepresentation of genes in developmental biology, cell cycle, and cytokine signaling in immune system pathways (Additional file 1: Table S5).

Next, we focused on 420 significant CpG sites correlated with clinical traits, which are more likely to have clinical relevance (Additional file 1: Table S4). We analyzed the 365 annotated genes to the 420 CpG sites using DAVID and MetaCore. In GO processes analyzed by DAVID we identified enrichment of regulation of transcription, RNA metabolic processes, cellular biosynthetic processes (Additional file 1: Table S6, *p* < FDR5%). This was further confirmed by GO analysis performed in MetaCore (Additional file 1: Table S7). Next, we analyzed gene-disease associations and identified enrichment of genes for pituitary ACTH hypersecretion, musculoskeletal abnormalities, and morbid obesity (FDR 5%, Additional file 1: Table S8). Transcription factors enrichment analysis revealed two highly active transcription factors, *TCF7L1* and *LMO2*, regulating 90 and 128 genes, respectively (Additional file 1: Table S9 and Additional file 1: Table S10). A look-up of all 4857 CpG sites in the eQTM dataset provided at https://www.genenetwork.nl/biosqtlbrowser/ showed that 38 CpG sites were associated with gene expression levels of 50 genes (Additional file 1: Table S11). Two CpG sites (cg13636880 and cg18888520) located within the genes *ZNF586* and *ZSCAN18*, respectively, were associated with gene expression of multiple zinc finger genes, which are known to be involved in regulation of transcription, DNA binding, and metal ion binding [45].

#### Replication of significant CpG sites associated with bariatric surgery

DNA methylation data from two independent cohorts was available for replication purposes. We looked up our top 4857 significant CpG sites in results of those two replication cohorts. The effect sizes, standard errors and *p* values can be found in Additional file 1: Table S12. Our findings could not be replicated in those two additional cohorts. This was confirmed in the meta-analysis, which combined results of all three cohorts. The combined effects were largely driven by our own results with high heterogeneity between cohorts (Additional file 1: Table S12, column U).

#### DNA methylation levels in patients undergoing bariatric surgery and the general population

In order to compare DNA methylation levels before and after bariatric surgery with the general population, DNA methylation data, obtained in the same experimental batch, was available for two healthy groups from the Lifelines cohort: a lean subgroup and subgroup that was considered healthy obese (*n* = 50 each, clinical characteristics are shown in Additional file 1: Table S13). The 1 year post-surgery levels of DNA methylation were found to be more similar to those observed for the healthy groups for all 4857 significant CpG sites, than the pre-surgical levels (*p* values < 2.2 × 10^−16^). This is illustrated by Additional file 2: Figure S6, which shows the methylation levels for the top 15 significant CpG sites from EWAS analysis in pre- and post-surgical patients and the two Lifelines subgroups.

#### Epigenetic age and epigenetic age acceleration before and after bariatric surgery

For all 40 patients, epigenetic age (EA) was calculated using the DNA methylation age calculator developed by Horvath. Before surgery, the mean EA was 3.17 years higher compared to the chronological age, while after surgery, the mean EA was 2.26 years higher compared to the chronological age (Table 3). Based on the individuals EA, the mean difference between pre- and post-surgery was 0.08 years, while chronologically, approximately one year had passed. Epigenetic age acceleration (EAA), commonly defined as the difference between EA and chronological age, was smaller after surgery (mean = − 0.92, *p* value = 0.039) suggesting significant improvement of biological age.

**Table 3:** Chronological age, epigenetic age (EA), and epigenetic age acceleration (EAA) pre- and post-surgery in severely obese patients

	Mean	SD	Min	Max
Chronological age, pre-surgery (years)	45.10	8.06	29	65
Chronological age, post-surgery (years)	46.10	8.06	30	66
EA, pre-surgery (years)	48.27	9.15	28.37	69.38
EA, post-surgery (years)	48.36	8.67	31.07	65.53
EA change	0.08	3.22	− 8.17	6.92
EAA, difference between epigenetic and chronological age pre-surgery	3.17	4.71	− 5.72	13.75
EAA, difference between epigenetic and chronological age post- surgery	2.26	5.05	− 7.47	17.28
Change in EAA	− 0.92^*^	0.34	− 1.75	3.53

*p value = 0.039

## Discussion

The aims of the current study were to identify the effects of bariatric surgery on three domains: clinical profile, DNA methylation, and biological age. We report significant changes in clinical profile at 3, 6, and 12 months after the surgery and in DNA methylation profile and biological age 12 months after surgery, suggesting that bariatric surgery has a prominent effect on individual health and epigenetics.

### Beneficial effects of bariatric surgery on weight and clinical profile

After bariatric surgery, we observed a sustained decrease in BMI, reflecting substantial weight loss in response to the intervention. A considerable decrease in BMI was also observed within the first year after the surgery in other similar studies [46–48]. In the CBS longitudinal cohort study, the peak weight decrease was reached 2 years after surgery and remained stable for at least 20 years [47]. Over 60% of patients included in our study underwent RYGB, which is the preferable method of bariatric surgery due to greater weight loss and higher diabetes remission rate (62% vs. 47% and 80.3% vs. 56.7%, respectively) compared to a gastric banding procedure [9, 49]. In our analysis, we combined all types of the surgery to increase the power of the study. As a sensitivity analysis, we repeated the analysis in the largest group (RYGB) and found similar results with less significant *p* values (all EWAS significant CpG sites, *n* = 4857, *p* values < 1 × 10^−4^; correlation of effect sizes between RYGB group and total group including all types of surgery was 0.99; data not shown).

Severe obesity is a known risk factor for type 2 diabetes and nearly 50% of type 2 diabetes patients are obese [50]. Risk of developing diabetes is even 20 times higher in obese than in lean individuals [51]. In line with these data, elevated levels of glucose, HbA1c, insulin, and HOMA-IR in the patients before the surgery suggest either pre-diabetic state or fully developed type 2 diabetes in 37.5% of severely obese patients. In our study, we observed improvement in post-operation glycemic profile: fasting glucose and insulin levels in blood were significantly lower over time within the first year after the surgery. The early improvement in glycemic traits observed in our study may be associated with caloric restriction leading to increased hepatic insulin sensitivity and improved beta cell function, also related to the post-bariatric high postprandial glucagon-like peptide 1 secretion [52]. Later, weight loss induces improvement in muscle insulin sensitivity, which enhances overall insulin sensitivity and glucose tolerance [53].

Hyperlipidemia, hypercholesterolemia, and hypertriglyceridemia associated with severe obesity are improved after bariatric surgery in at least 70% of patients [9]. In our data, we observed similar changes in levels of triglycerides, total, and LDL cholesterol levels after the surgery.

With severe obesity progressing, adipose tissue becomes more dysfunctional. Increased production of pro-inflammatory cytokines in adipose tissue contributes to systemic inflammation, which is a hallmark of severe obesity. Elevated FFAs increase insulin secretion in the pancreas and decreases insulin sensitivity in the liver and muscle, which contributes to obesity-related metabolic complications [54]. C-reactive protein was significantly lower after bariatric surgery, further confirming reduced inflammation after weight loss.

### Epigenetic changes after bariatric surgery

The beneficial effect of bariatric surgery on metabolic and body composition parameters observed in our study paralleled a shift in DNA methylation patterns in blood. As shown previously, DNA methylation levels in blood can (partly) reflect epigenetic signatures in target tissues for metabolic diseases such as the adipose tissue, liver, and muscle [55–57]. Although studies investigating the effect of bariatric surgery on DNA methylation levels on a genome-wide level are scarce, we identified one study in the adipose tissue and one study in blood. In the first study, fifteen obese women with similar age distribution to our study underwent gastric bypass surgery and another surgery with a mean follow-up of 17.5 months. The analysis revealed differentially methylated CpG sites in omental (15 CpG sites, Bonferroni corrected *p* value) and subcutaneous adipose tissue (3601 CpG sites, Bonferroni corrected *p* value), with higher methylation at most significant CpG sites before the surgery [58]. In the second study in blood, however, the direction of differential methylation was different, 666 CpG sites showed higher methylation after surgery in 24 women who underwent RYGB (a mean follow-up 6 months) [35]. Another intervention study investigating the effect of physical exercise on DNA methylation levels in adipose tissue found 17,975 differentially methylated individual CpG sites (based on FDR < 5%), of which 16,470 are with higher methylation and 1505 are with lower methylation in response to 6 months of exercise in 23 non-obese men (BMI < 30) [59]. Similar to our observations, the effects of intervention, although to a lesser degree than in our study, were weight loss and significant decrease in waist circumference and waist to hip ratio. We hypothesize that weight reduction and related metabolic and inflammatory changes occurring initially after the surgery lead to altered DNA methylation levels, similar to what has been shown in an obesity EWAS using Mendelian randomization methods, where changes in DNA methylation levels were likely to be due to changes in BMI [16].

DNA methylation levels at all significant CpG sites were higher after bariatric surgery. However, the unidirectional (hyper) methylation changes were somewhat unexpected. The results are also unlikely due to a potential technical bias, since all the steps including DNA isolation, sample randomization on the plates, bisulfite conversion, and the hybridization to the chip were performed within the same experimental batch. Interestingly, folic acid deficiency has been reported among bariatric patients after surgery [60]. Folic acid is the donor of methyl group, required for DNA methylation reactions in vivo [61]. Changes in DNA methylation may partly deplete folate reserves after bariatric surgery and explain this so-called Great Folate Mystery in bariatric patients post-surgically together with decreased food intake. This hypothesis needs further investigation.

Twelve months after the bariatric surgery, DNA methylation levels were changed at numerous CpG sites. Epigenetic mechanisms are modifiable via environmental exposures and lifestyle factors, therefore, such a considerable change after bariatric surgery may have an impact on DNA methylation [62]. In this regard, it was proposed that epigenetic regulation could mediate the benefit of bariatric surgery on body weight and the metabolic disturbances associated with excess body weight, such as insulin resistance, hypertension, and cardiovascular disease [12].

Accordingly, adjusting our analysis for BMI, glucose, insulin, and CRP changes decreased the number of significant CpG sites, suggesting that post-operation changes in clinical profile can explain part of the association between bariatric surgery and DNA methylation. We conclude that DNA methylation changes observed after bariatric surgery are a result of clinical changes, including but not limited to weight loss.

We also compared the pre- and post-surgery DNA methylation levels at top significant CpG sites with DNA methylation levels in healthy individuals. Our data suggests restoration of the “severe obesity” DNA methylation patterns 12 months after surgery to the epigenetic profiles found in general populations. In agreement with these observations, DNA methylation levels of the SCD gene promoter were found to be lower in morbidly obese subjects (*n* = 120) before bariatric surgery but increased 6 months after RYGB to similar levels as in the control group (*n* = 30, BMI similar to after surgery group) [63].

### Potential functional relevance of bariatric surgery-associated methylation

The 420 CpG sites significantly correlated with metabolic traits may represent clinically relevant changes in DNA methylation. The TF-set enrichment analysis of those CpG sites annotated to gene names revealed two interesting transcription factors: TCF7L1 and LMO2. TCF7L1 is a transcription factor 7 like 1 involved in the regulation of cell cycle. It belongs to the same family as the well-known type 2 diabetes susceptibility gene transcription factor 7 like 2 (TCF7L2) found in multiple GWAS studies [64]. Additionally, TCF7L2 was associated with type 2 diabetes in epigenetic studies performed in blood and pancreas [65, 66]. Proteins of TCF7L1 and TCF7L2 show similar features and are important for the regulation of Wnt/β-catenin signaling during adipocyte development [67, 68]. The LMO2 (LIM Domain Only 2) protein has a crucial role in hematopoietic development and is associated with leukemia [69]. Further studies are needed to elucidate the role of the epigenetic regulation of LM02 in the field of obesity and metabolic disorders.

To the best of our knowledge, this is the first study which shows an impact of bariatric surgery on biological (epigenetic) age. The only other study we identified was conducted in the liver tissue which showed that despite a rapid decrease in BMI in a 9-month period, the epigenetic age was not reversed [70]. Although we observed a minor improvement in biological age after surgery, the biological age of the patients studied remained increased compared to the chronological age. Other studies have shown BMI to be associated with increased EAA in blood and buccal cells [24, 71]. In our study, the EAA, as expected, significantly decreased upon surgery, suggesting improvement of biological age together with improvement of clinical factors after bariatric surgery.

### Strengths and limitations

The strength of our study is its longitudinal setting, where bariatric patients were examined at multiple time points. Due to the dynamic nature of epigenetic markers, the EWAS analysis was conducted at 2 time points (before and 12 months after the surgery) to unravel the effect of the bariatric surgery on the DNA methylation profile. Although we realized that the follow-up period of 12 months is relatively short, we still observed major changes in clinical and epigenetic profile. Many other studies focused on women, while our population was mixed, making it possible to extend those findings to males. Using blood as a tissue for conducting EWAS is a limitation in our study, however, metabolically active tissues are difficult to obtain. Additionally, we adjusted our analysis for blood cell types, knowing that DNA methylation can vary per cell type. We also list a relatively small sample size as another limitation of our study. Unfortunately, we were not able to replicate our findings in two independent cohorts. We observed high heterogeneity in effect sizes between cohorts, which may be related to shorter follow-up time (6 instead of 12 months), substantially smaller sample sizes and power (40 vs. 24 and 11) or other design differences between discovery and replication studies. Interestingly, DNA methylation patterns after the surgery were more similar to general population levels, suggesting a restoration of more healthy DNA methylation levels 12 months after the surgery.

## Conclusions

In summary, our study shows that bariatric surgery leads to improvement of clinical outcomes, including substantial decrease in BMI, as well as to epigenetic changes. Such drastic intervention may restore DNA methylation profiles in patients with severe obesity towards the patterns observed in healthy subjects. The beneficial effect of bariatric surgery on changes in DNA methylation markers could be mediated by body weight and metabolic parameters and in the future, those markers may be useful for obesity-related comorbidities.

## Supplementary information

**Additional file 1: Table S1.** Type of the bariatric surgery in 40 severely obese patients. **Table S2.** Baseline and follow-up characteristics of severely obese cohort before and 12 months after bariatric surgery. **Table S3.** 4857 significant CpG sites associated with pre- and post bariatric surgery difference form EWAS analysis. **Table S4.** Correlations of change in DNA methylation with change in clinical measures in pre- and post-surgery cohort. **Table S5.** Reactome enriched terms based on EWAS results. **Table S6.** Gene ontology terms enriched based on correlated with clinical traits CpG sites from DAVID software. **Table S7.** Top 10 gene ontology terms enriched based on correlated with clinical traits CpG sites from Metacore software. **Table S8.** Top 10 gene-dieseses assotiations enriched based on correlated with clinical traits CpG sites from Metacore software. **Table S9.** Top 30 trancription factors calculated based on a correlated CpG sites with clinical traits by Metacore software. **Table S10.** TCF7L1 and LMO2 transcription factore and their associated genes from Metacore software. **Table S11.** DNA methylation levels significantly associated with gene expression levels from blood derived from publically available data (https://www.genenetwork.nl/biosqtlbrowser/). **Table S12.** 4857 significant CpG sites associated with pre-and post bariatric surgery difference from EWAS analysis separately for 3 cohorts and a combined effect. **Table S13.** Characteristics of 2 Lifelines subcohorts, healthy lean and healthy obese.

**Additional file 2: Figure S1.** Volcano plot showing significant CpG sites (in red) from EWAS analysis on pre- and 12 months post-surgical differences in methylation levels from severely obese patients. **Figure S2.** Manhattan plot of EWAS analysis on pre- and 12 months post-surgical differences in methylation levels from severely obese patients. **Figure S3.** Location of significant CpG sites from EWAS analysis on pre- and 12 months post-surgical differences in methylation levels from severely obese patients compared to total CpG sites from Illumina array. Top plots represents CpG sites location in relation to the gene, bottom plots shows CpG sites location in relation to the CpG islands. (Abbreviations: TSS1500 -200–1500 bases upstream of the transcription start site, TSS200 – up to 200 bases upstream of the transcription start site, ***- significant p-values<2.2x10-16). **Figure S4.** Boxplots of estimated white cell types distributions before and 12 months after surgery. (*p*-values: * <0.05, **<0.01, ***<0.001). **Figure S5.** Venn diagram of significant CpG sites from sensitivity analysis adjusted for clinical changes after bariatric surgery (n=30). **Figure S6.** DNA methylation levels for top 15 CpG sites in severely obese patients before and after surgery and in sub-cohorts from Lifelines.

## Data Availability

The datasets used and/or analyzed during the current study are available from the corresponding author on reasonable request.

## Abbreviations

ALAT: Alanine transaminase
ASAT: Aspartate transaminase
BMI: Body mass index
CRP: C-reactive protein
CpG sites: Cytosine-guanine dinucleotides
EA: Epigenetic age
EAA: Epigenetic age acceleration
FFA: Free fatty acids
GO: Gene ontology
KEGG: Kyoto Encyclopedia of Genes and Genomes
RYGB: Roux-and-Y gastric bypass
TG: Triglycerides
