# Supplementary material for: The effects of bariatric surgery on clinical profile, DNA methylation, and ageing in severely obese patients
Source: Clin Epigenetics. 2020 Jan 20;12:14. doi: 10.1186/s13148-019-0790-2 (PMC6972025; doi:10.1186/s13148-019-0790-2)

Additional file 2: Figure S1. Volcano plot showing significant CpG sites (in red) from EWAS analysis on pre- and 12 months post-surgical differences in methylation levels from severely obese patients.
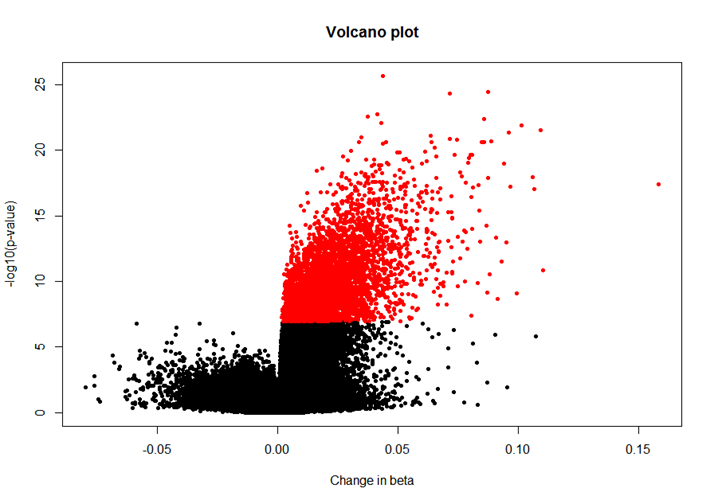


Additional file 2: Figure S2. Manhattan plot of EWAS analysis on pre- and 12 months post-surgical differences in methylation levels from severely obese patients.


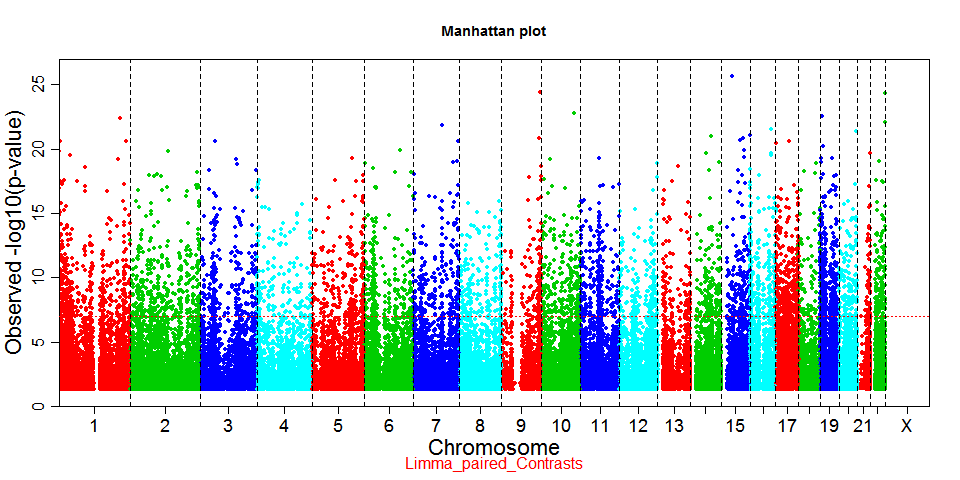


Additional file 2: Figure S3. Location of significant CpG sites from EWAS analysis on pre- and 12 months post-surgical differences in methylation levels from severely obese patients compared to total CpG sites from Illumina array. Top plots represents CpG sites location in relation to the gene, bottom plots shows CpG sites location in relation to the CpG islands. (Abbreviations: TSS1500 -200–1500 bases upstream of the transcription start site, TSS200 – up to 200 bases upstream of the transcription start site, ***- significant *p-*values<2.2x10^-16^).


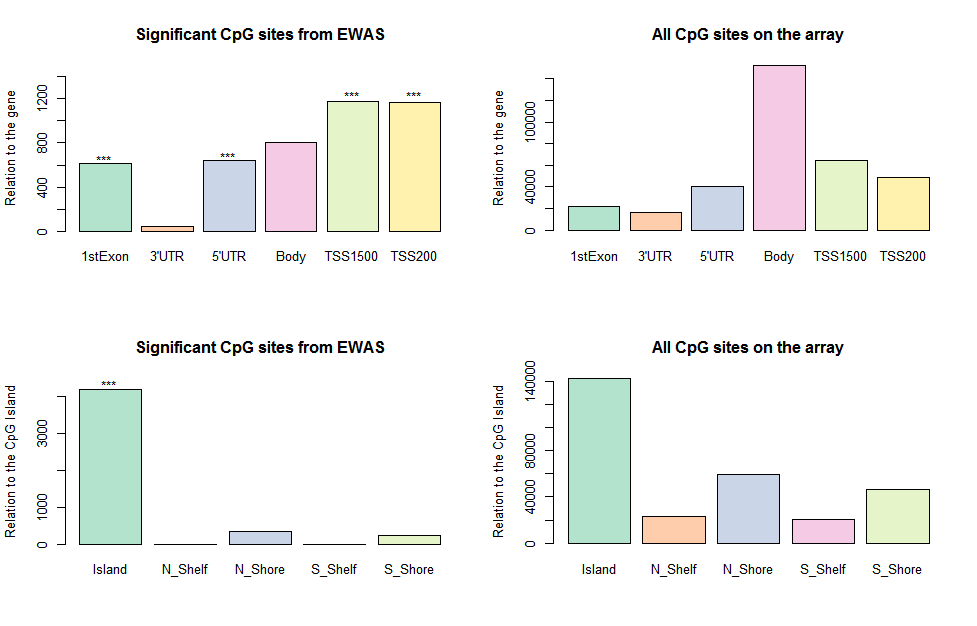


Additional file 2: Figure S4. Boxplots of estimated white cell types distributions before and 12 months after surgery. (p-values: * <0.05, **<0.01, ***<0.001)


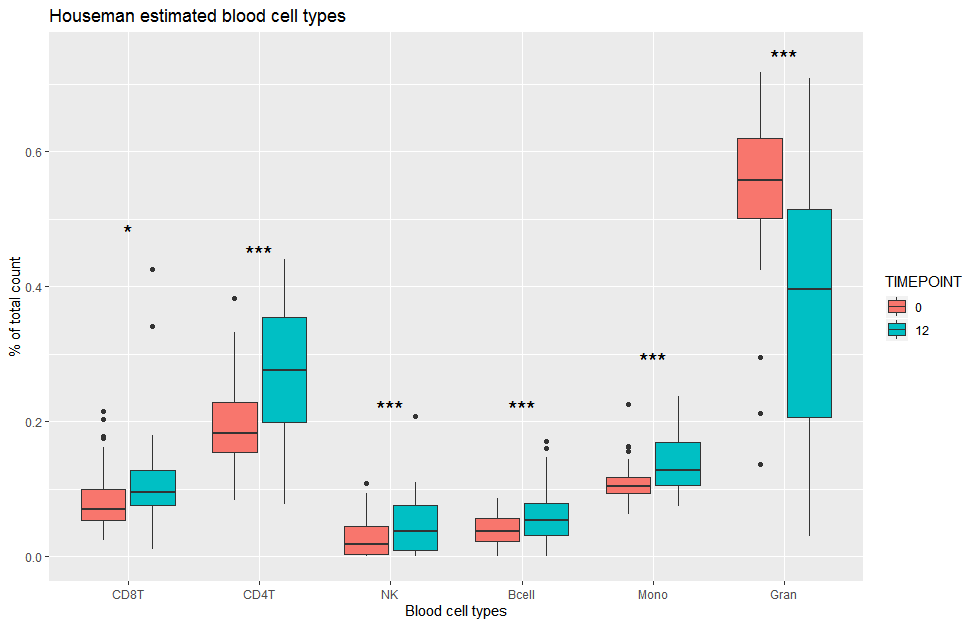


Additional file 2: Figure S5. Venn diagram of significant CpG sites from sensitivity analysis adjusted for clinical changes after bariatric surgery (n=30).


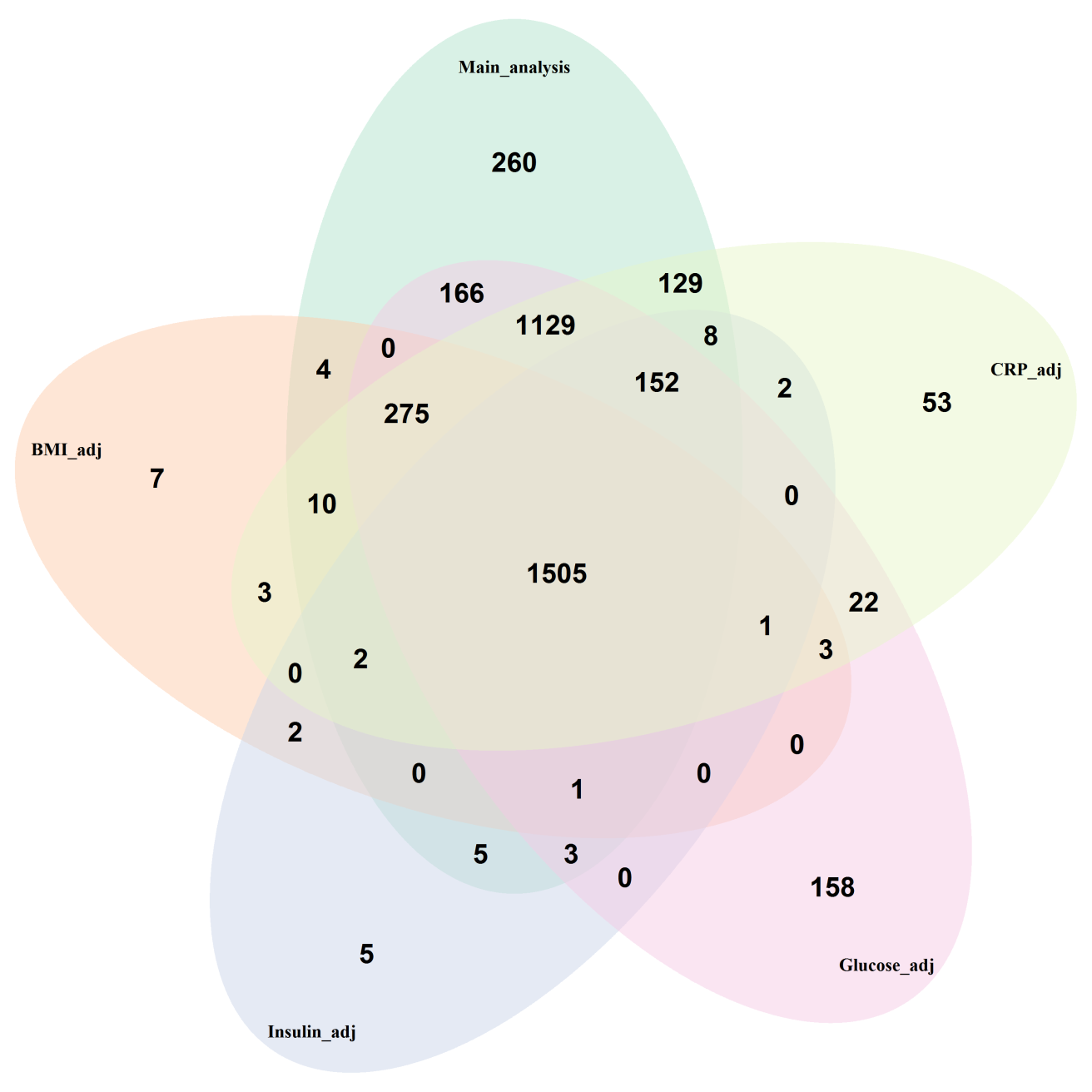


Additional file 2: Figure S6. DNA methylation levels for top 15 CpG sites in severely obese patients before and after surgery and in sub-cohorts from Lifelines.


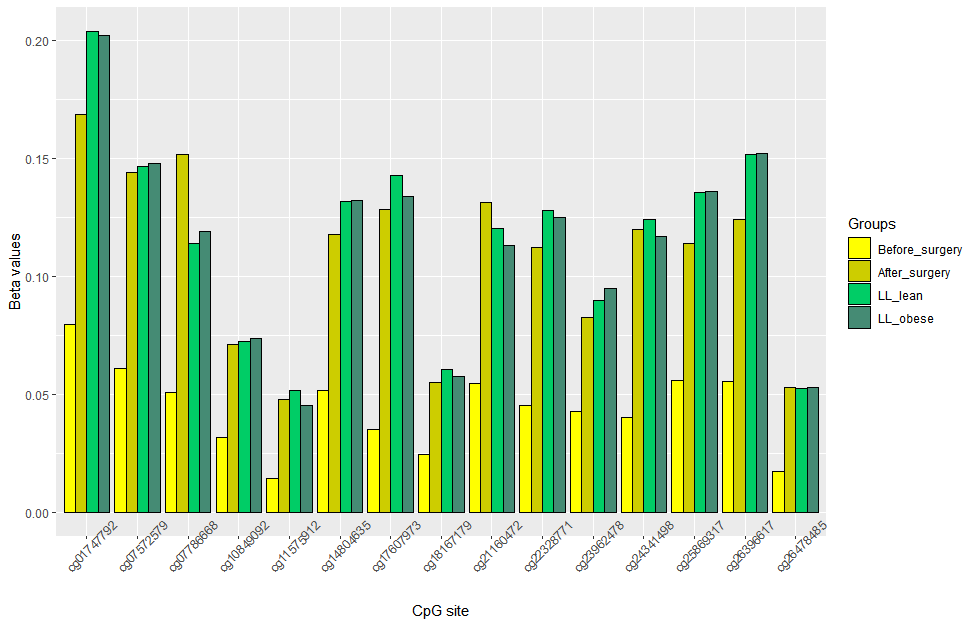

Supplement: Supplementary file 2 — Additional file 2: Figure S1. Volcano plot showing significant CpG sites (in red) from EWAS analysis on pre- and 12 months post-surgical differences in methylation levels from severely obese patients. Figure S2. Manhattan plot of EWAS analysis on pre- and 12 months post-surgical differences in methylation levels from severely obese patients. Figure S3. Location of significant CpG sites from EWAS analysis on pre- and 12 months post-surgical differences in methylation levels from severely obese patients compared to total CpG sites from Illumina array. Top plots represents CpG sites location in relation to the gene, bottom plots shows CpG sites location in relation to the CpG islands. (Abbreviations: TSS1500 -200–1500 bases upstream of the transcription start site, TSS200 – up to 200 bases upstream of the transcription start site, ***- significant p-values<2.2x10^-16^). Figure S4. Boxplots of estimated white cell types distributions before and 12 months after surgery. (p-values: * <0.05, **<0.01, ***<0.001). Figure S5. Venn diagram of significant CpG sites from sensitivity analysis adjusted for clinical changes after bariatric surgery (n=30). Figure S6. DNA methylation levels for top 15 CpG sites in severely obese patients before and after surgery and in sub-cohorts from Lifelines. [file 13148_2019_790_MOESM2_ESM.docx]
